# IonchanPred 2.0: A Tool to Predict Ion Channels and Their Types

**DOI:** 10.3390/ijms18091838

**Published:** 2017-08-24

**Authors:** Ya-Wei Zhao, Zhen-Dong Su, Wuritu Yang, Hao Lin, Wei Chen, Hua Tang

**Affiliations:** 1 Key Laboratory for Neuro-Information of Ministry of Education, School of Life Science and Technology, Center for Informational Biology, University of Electronic Science and Technology of China, Chengdu 610054, China; lianyingteng@hotmail.com (Y.-W.Z.); zhendong_su@163.com (Z.-D.S.); wyang@imu.edu.cn (W.Y.); 2 Development and Planning Department, Inner Mongolia University, Hohhot 010021, China; 3 Department of Physics, School of Sciences, and Center for Genomics and Computational Biology, North China University of Science and Technology, Tangshan 063000, China; 4 Department of Pathophysiology, Southwest Medical University, Luzhou 646000, China

**Keywords:** ion channels, pseudo-dipeptide composition, machine learning method

## Abstract

Ion channels (IC) are ion-permeable protein pores located in the lipid membranes of all cells. Different ion channels have unique functions in different biological processes. Due to the rapid development of high-throughput mass spectrometry, proteomic data are rapidly accumulating and provide us an opportunity to systematically investigate and predict ion channels and their types. In this paper, we constructed a support vector machine (SVM)-based model to quickly predict ion channels and their types. By considering the residue sequence information and their physicochemical properties, a novel feature-extracted method which combined dipeptide composition with the physicochemical correlation between two residues was employed. A feature selection strategy was used to improve the performance of the model. Comparison results of in jackknife cross-validation demonstrated that our method was superior to other methods for predicting ion channels and their types. Based on the model, we built a web server called IonchanPred which can be freely accessed from http://lin.uestc.edu.cn/server/IonchanPredv2.0.

## 1. Introduction

**Figure 1:**
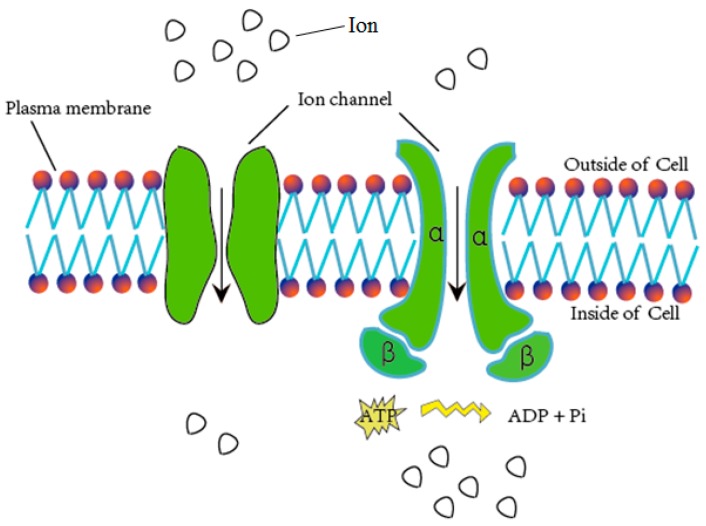
Schematic diagram of material exchange through ion channels.

Ion channels are pore-forming membrane proteins for the transmembrane exchange of inorganic ions (as shown in Figure 1). Ion channels exist in the membranes of all cells and are required in numerous physiological and pathological processes, such as regulating neuronal and cardiac excitability, muscle contraction, hormone secretion, fluid movement, and immune cell activation [1]. Due to their important role in biological processes, ion channels are often used as targets for disease diagnosis and drug development. There are over 300 types of ion channels in living cells [2], and they differ in their structure and function. According to the different gating mechanisms, the ion channels can be mainly divided into two categories, namely voltage-gated ion channels (VGIC) and ligand-gated ion channels (LGIC) [3]. The opening and closing of the voltage-gated ion channels depends on the change of the membrane potential, whereas the state of the ligand channels is closely related to the binding of the ligand. The voltage-gated ion channels can be further classified into the following four subclasses: potassium (K^+^), sodium (Na^+^), calcium (Ca^2+^), and anion channels.

In view of the important role and multiple types of ion channels, the structures and functions of ion channels have continued to attract the attention of numerous researchers in recent years [4,5,6,7,8,9,10]. Due to the rapid growth of proteomic data, it is particularly important to develop bioinformatics tools to quickly predict and identify ion channels and their types. Consequently, many computational methods based on machine learning algorithm have been developed in the last 10 years [11,12,13,14,15,16,17]. Liu et al. [11] proposed a method to identify voltage-gated potassium channels, and indicated that the local sequence information-based method was better than the global sequence information-based method. Saha et al. [12] developed a support vector machine (SVM)-based method by using amino acid composition and dipeptide composition to predict voltage-gated ion channels and their subtypes. In 2011, our group [13] developed a more generalized predictive tool, called IonchanPred, and identified ion channels and their types accurately. Recently, Tiwari et al. [16] proposed a random forest based methods and Gao et al. [17] proposed a model to predict ion channels and their subfamilies by combining a SVM-based model with BLAST sequence similarity search. Although many predictors for identifying ion channels are available, three essential issues remain elusive. Firstly, the use of high similarity sequences may overestimate the performance of a model. Secondly, the long-range effect is lost in most published models. Thirdly, web servers should be improved.

**Figure 2:**
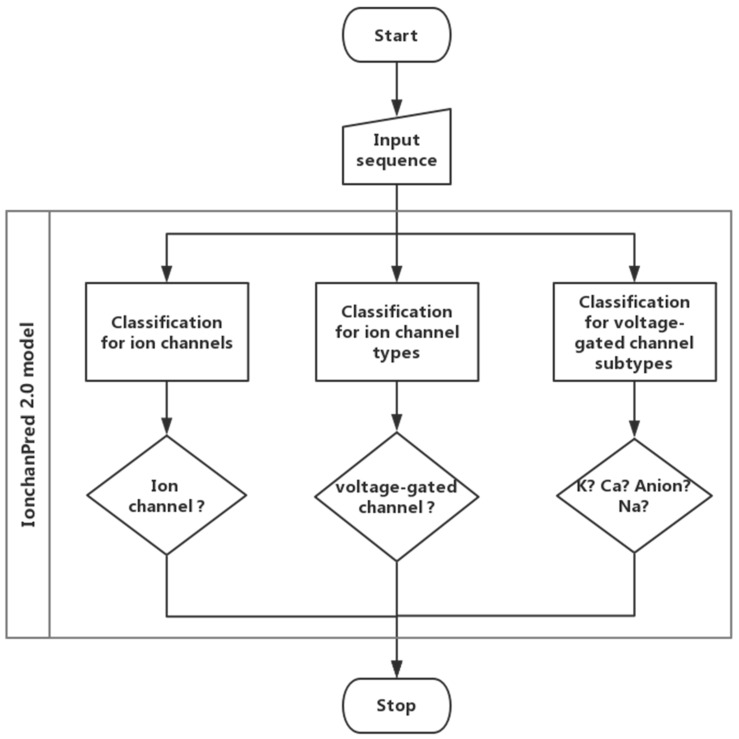
Workflow of the IonchanPred 2.0 model.

In this paper, a support vector machine-based model was constructed to quickly identify ion channels and their types. In this model, a novel feature extraction method called pseudo-dipeptide composition was employed. The analysis of variance (ANOVA) [18] was introduced to rank features. The incremental feature selection (IFS) was employed to find an optimized feature set which can produce the maximum accuracy. Finally, a web server called IonchanPred 2.0 was established. The flow chart is shown in Figure 2.

## 2. Results and Discussion

### 2.1. Parameter Optimization

The establishment of our proposed model depends on two important parameters: λ and ω. λ factor denotes the rank of correlation and the larger λ may contain more global sequence-order information. ω represents the weight of the correlation of residues’ physiochemical properties compared to the traditional dipeptide component. To obtain the optimal value for the two parameters, a serial of experiments was performed according to the following standard:

(1) $$\{\begin{array}{c} 1\leq \lambda \leq 30\;\mathrm{with}\;\mathrm{step}\;\Delta =1\\ 0.05\leq \omega \leq 0.70\;\mathrm{with}\;\mathrm{step}\;\Delta =0.05 \end{array}$$

In view of this, a total of 30×14=420 individual combinations were obtained. Then, we can investigate the accuracy of SVM with the jackknife test. The optimal parameter combinations corresponding to the three individual datasets are shown in Table 1. It shows that the highest overall accuracy can be up to 87.5% when λ=21 and ω=0.20 for the dataset including ion channels and non-ion channels (NIC). For the benchmark dataset VGIC vs. LGIC, the maximum accuracy is 93.9% when λ=7 and ω=0.30. The best model for four types of VGIC prediction can produce overall accuracy of 89.1%. After the parameters are optimized, the samples for the three individual datasets can be respectively formulated as follows: a 589-dimensional vector involving 400 dimensions for traditional dipeptide composition and 9×21=189 dimensions for correlation information for IC vs. NIC prediction, a vector involving 400+9×7=463 dimensions for VGIC vs. LGIC, and a vector involving 400+9×9=481 dimensions for four types of voltage-gated ion channels datasets.

### 2.2. Model Establishment

**Figure 3:**
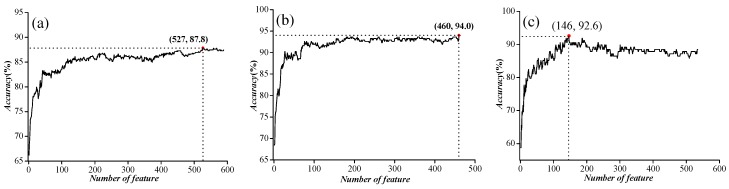
The feature selection results for three independent datasets. (**a**) Incremental feature selection (IFS) curve for ion channel (IC) vs. non-ion channel (NIC) prediction; (**b**) IFS curve for voltage-gated ion channels (VGIC) vs. ligand-gated ion channels (LGIC) prediction; (**c**) IFS curve for four types of VGIC prediction.

In order to further improve the accuracy, we used ANOVA to exclude noise or redundant information. After the feature selection, the features were sorted according to the decreasing order of the *F* values described in Section 3.3 *Feature* *Selection* to obtain the feature list. Then, we used the IFS to determine the optimal number of features, as described below. The feature subset starts from a feature ranking first in the feature list. A new feature subset was composed when the second feature of this list was added. We repeated this process until all candidate features were added. In this case, we obtained 589, 463, and 535 feature subsets, respectively, for the three benchmark datasets mentioned above. The performance of each feature subset was examined by using SVM with the jackknife test. We plotted the relationship between the overall accuracy and the numbers of features in Figure 3. We noticed that the prediction performances were the best when the top ranked 527, 460, and 147 features were used for the three datasets, respectively.

In order to further evaluate the predictive performance of our model, we also calculated the average accuracies for the three datasets. A comparison of the results with the previous model [13] are shown in Table 2. It is clear that the predictive performance of our proposed model is better than the previous model.

## 3. Materials and Methods

### 3.1. Benchmark Databases

The data used to establish the prediction model in this paper were collected from Lin et al. [13]. The sequences of ion channels were collected from the Universal Protein Resource (UniProt) [19] and the Ligand-Gated Ion channel database [20]. To construct a high-quality benchmark dataset, some sequences were removed according to three characteristics. Firstly, a sequence that contained some ambiguous residues (such as “X”, “B”, “Z”). Secondly, a sequence that was the fragment of other proteins. Thirdly, a sequence that was annotated based on homology or prediction. Then, redundant sequences were removed by using the CD-HIT [21] program with a sequence identity threshold of 40%, which has been widely used to filter out redundant samples in genomics and proteomics [22,23,24,25,26].

After the raw data were preprocessed, we finally obtained 298 ion channels including 148 voltage-gated ion channels and 150 ligand-gated ion channels. These voltage-gated ion channels can be classified into four subtypes as follows: 81 potassium (K^+^), 29 calcium (Ca^2+^), 12 sodium (Na^+^), and 26 voltage-gated anion channels. Here, all the 300 non-ion channel proteins were randomly selected from the membrane proteins which were not marked as ion channels in the UniProt database. Moreover, any two sequences in these non-ion channels should guarantee that the identity between them is less than 40%.

### 3.2. Feature Extraction of Samples

In order to characterize each protein sequence as accurately as possible, the order effect of sequence was usually selected as a method for generating effective feature vectors. Therefore, PseAAC [27,28] incorporating dipeptide composition was selected as the method for feature extraction of protein samples in this paper.

Assuming that there is a protein sequence of *L* amino acid residues:

(2) $$Ρ=R_{1}R_{2}R_{3}R_{4}R_{5}R_{6}R_{7}\ldots R_{L}$$

where $R_{i}\left(i=1,2,3\ldots L\right)$ represents the amino acid residue at i-th sequence position. Therefore, we can get a set of feature vectors with the dimension of 400+nλ from any sequence like Equation (1)

(3) $$Ρ={\left[P_{1},P_{2},\ldots ,P_{400},P_{401},\ldots ,P_{400+n\lambda }\right]}^{T}$$

where the first 400 features $P_{1},P_{2},\ldots ,P_{400}$ represent the effect of the classical dipeptide composition; the nλ elements $P_{400+1},P_{400+2},\ldots ,P_{400+n\lambda }$ in addition to the 400 components represent the sequence order effect of protein samples, namely the first tier to λ-th tier correlation factors of protein sequence. These features can be calculated by:

(4) $$P_{u}=\{\begin{array}{l} \frac{f_{u}}{\sum_{i=1}^{400}f_{i}+\omega \sum_{j=1}^{n\lambda }\tau_{j}}\;\left(1\leq u\leq 400\right)\\ \frac{\omega \tau_{u}}{\sum_{i=1}^{400}f_{i}+\omega \sum_{j=1}^{n\lambda }\tau_{j}}\;\left(400+1\leq u\leq 400+n\lambda \right) \end{array}$$

where $f_{i}\left(i=1,2,\ldots ,400\right)$ is the normalized occurrence frequencies of the 400 dipeptides in protein **P**; ω is the weight factor; $\tau_{j}\;\left(j=1,2,\ldots ,n\lambda \right)$ is the *j*-tier sequence-correlation factor computed by:

(5) $$\left\{ \begin{array}{c} \tau_{1}=\frac{1}{L-1}\overset{L-1}{\underset{i=1}{\sum }}H_{i,i+1}^{1}\\ \tau_{2}=\frac{1}{L-1}\overset{L-1}{\underset{i=1}{\sum }}H_{i,i+1}^{2}\\ \ldots \\ \tau_{n}=\frac{1}{L-1}\overset{L-1}{\underset{i=1}{\sum }}H_{i,i+1}^{n}\\ \tau_{n+1}=\frac{1}{L-2}\overset{L-2}{\underset{i=1}{\sum }}H_{i,i+2}^{1}\\ \tau_{n+2}=\frac{1}{L-2}\overset{L-2}{\underset{i=1}{\sum }}H_{i,i+2}^{2}\\ \ldots \\ \tau_{2n}=\frac{1}{L-2}\overset{L-2}{\underset{i=1}{\sum }}H_{i,i+2}^{n}\\ \ldots \\ \tau_{n\lambda -1}=\frac{1}{L-\lambda }\overset{L-\lambda }{\underset{i=1}{\sum }}H_{i,i+\lambda }^{n-1}\\ \tau_{n\lambda }=\frac{1}{L-\lambda }\overset{L-\lambda }{\underset{i=1}{\sum }}H_{i,i+\lambda }^{n} \end{array}\right.$$

where $H_{i,j}^{n}$ is the correlation function of physicochemical properties and can be calculated as:

(6) $$H_{i,j}^{n}=h^{n}\left(R_{i}\right)\cdot h^{n}\left(R_{j}\right)$$

where $h^{n}\left(R_{i}\right)$ denotes the value of n-th kind physicochemical property of $R_{i}$; $h^{n}\left(R_{j}\right)$ is similar. To obtain the high-quality feature set, all the data of physicochemical properties must be subjected to a standard conversion as below:

(7) $$h^{k}\left(R_{i}\right)=\frac{h_{0}^{k}\left(R_{i}\right)-\sum_{\alpha =1}^{20}h_{0}^{k}\left(R_{\alpha }\right)/20}{\sqrt{\sum_{u=1}^{20}{\left[h_{0}^{k}\left(R_{i}\right)-\sum_{\alpha =1}^{20}h_{0}^{k}\left(R_{\alpha }\right)/20\right]}^{2}}}$$

where $R_{i}\left(i=1,2,\ldots ,20\right)$ represents the 20-native amino acid according to the alphabetical order of their single-letter codes: A, C, D, E, F, G, H, I, K, L, M, N, P, Q, R, S, T, V, W, and Y. $h_{0}^{k}\left(R_{i}\right)$ denotes the original value of the k-th physicochemical property for residue $R_{i}$. The values of each physicochemical property obtained after the standard conversion have two advantages. These values will have a zero-mean over the 20 native amino acids and remain unchanged if they are subjected to the same conversion procedure again. The values of the nine kinds of physicochemical properties used in this paper are from previous results [29].

### 3.3. Feature Selection

Generally, all features do not equally contribute to an ion channel prediction system. Some features make key contributions, whereas some others make minor contributions [30,31]. Therefore, the selection of features is an important step for establishing an effective prediction model. To analyze these feature vectors, ANOVA was used to choose the optimal feature sets in this paper.

In order to assess the contribution of each feature to the predictive system, the *F* value was defined as follows:

(8) $$F\left(\lambda \right)=\frac{S_{B}^{2}\left(\lambda \right)}{S_{W}^{2}\left(\lambda \right)}$$

where $S_{B}^{2}\left(\lambda \right)$ and $S_{W}^{2}\left(\lambda \right)$ respectively denote the sample variance between groups (also called means square between, MSB) and the sample variable within groups (also called means square within, MSW), and are expressed as:

(9) $$\{\begin{array}{l} S_{B}^{2}(\lambda )=\frac{\sum_{i=1}^{K}n_{i}{\left(\sum_{j=1}^{n_{i}}f_{ij}(\lambda )/n_{i}-\sum_{i=1}^{K}\sum_{j=1}^{n_{i}}f_{ij}(\lambda )/\sum_{i=1}^{K}n_{i}\right)}^{2}}{K-1}\\ S_{W}^{2}(\lambda )=\frac{\sum_{i=1}^{K}\sum_{j=1}^{n_{i}}{\left(f_{ij}(\lambda )-\sum_{i=1}^{K}\sum_{j=1}^{n_{i}}f_{ij}(\lambda )/\sum_{i=1}^{K}n_{i}\right)}^{2}}{N-K} \end{array}$$

where *K* and *N* respectively denote the number of groups and the total number of samples. $f_{ij}\left(\lambda \right)$ represents the frequency of the λ-th feature of the j-th sample in the i-th group. $n_{i}$ denotes the total number of samples in the i-th group. Thus, each feature corresponds to an *F* score.

Obviously, the larger *F* value means the greater contribution of the corresponding feature to the classification. Thus, according to their *F* values, we may rank all features. Subsequently, we used the incremental feature selection (IFS) to determine the optimal number of features [32]. Firstly, we examined the accuracy of the first feature subset including a feature with the highest *F* value in the ranked feature set. Secondly, we investigated the accuracy of the second feature subset which was produced by adding the feature with the second highest *F* value. This process was repeated from the higher *F* to the lower *F* value until all candidate features were added. The performances of all feature subsets were evaluated. Then, we were able to obtain the best feature subset which was capable of producing the maximum accuracy.

### 3.4. Support Vector Machine

SVM is a kind of classification algorithm that can improve the generalization ability of machine learning and achieve the minimization of experience risk and confidence scope by minimizing the structural risk. Therefore, a good statistical result can be usually achieved even using a small sample. SVM, as a powerful supervised learning method, has been widely used in various fields including bioinformatics [33,34,35,36,37,38]. In this paper, we used LIBSVM 3.21 [39] which could be freely downloaded from http://www.csie.ntu.edu.tw/~cjlin/libsvm/. The radial basis function (RBF) kernel was selected as kernel function and one vs. one (OVO) strategy was used for multiclass classification. For achieving the optimal model, the penalty constant C and the kernel width parameter λ were tuned by an optimization procedure with a grid search method [39]. The search spaces for *C* and λ were [$2^{-5},2^{15}$] and [$2^{5},2^{-15}$] with steps being 2 and$\;2^{-1}$, respectively.

### 3.5. Performance Evaluation

A cross-validation technique is generally employed to estimate the accuracy of a predictive model. Three cross-validation methods including the independent dataset test, subsampling test, and jackknife test can be used [40,41,42,43]. Among them, the jackknife test is considered to be the most objective and rigorous one. Therefore, the jackknife test was employed to assess the performance of our methods.

In addition, we also used other assessment criteria to evaluate the effectiveness of our predictive model in this paper. These assessment criteria, including sensitivity (*Sn*), overall accuracy (*OA*), and average accuracy (*AA*), are defined as follows:

(10) $$S_{n}\left(i\right)=\frac{TP_{i}}{TP_{i}+FN_{i}}$$

(11) $$OA=\sum_{i=1}^{n}\frac{TP_{i}}{N}$$

(12) $$AA=\sum_{i=1}^{n}\frac{S_{n}\left(i\right)}{n}$$

where $TP_{i}$ and $FN_{i}$ respectively denote true positives and false negatives of the i-th class. *N* and *n* represent the total number of samples and number of classes, respectively.

## 4. Conclusions

We constructed an SVM-based model for the accurate prediction of ion channel proteins and their types. In this model, a pseudo-dipeptide composition was adopted to extract features. The ANOVA was used to exclude noise or redundant information of feature vectors and then IFS was employed to determine the optimal number of features. High accuracies indicated that the proposed method was an effective tool for predicting ion channels and their types. A free web server based on the proposed method presented in this paper has been constructed and is accessible at the website (http://lin.uestc.edu.cn/server/IonchanPredv2.0).

## Figures and Tables

**Table 1:** Optimal parameters for the three datasets.

Database	*λ*	*ω*	*OA* (%)
IC vs. NIC	21	0.20	87.5
VGIC vs. LGIC	7	0.30	93.9
four types of VGIC	9	0.15	89.1

IC: ion channels; NIC: non-ion channels; VGIC: voltage-gated ion channels; LGIC: ligand-gated ion channels; *OA*: overall accuracy.

**Table 2:** Performance evaluation parameters of our proposed model and a previous model.

Datasets		Our Model			Previous Model [13]		
		*Sn*	*OA*	*AA*	*Sn*	*OA*	*AA*
IC vs. NIC	IC	80.2	87.8	87.8	85.9	86.6	86.6
	NIC	95.3			87.3		
VGIC vs. LGIC	VGIC	94.7	94.0	94.0	94.6	92.6	92.7
	LGIC	93.2			90.7		
Types of VGIC	K^+^	97.5	92.6	87.7	92.6	87.8	83.7
	Ca^2+^	89.7			82.8		
	Na^+^	75.0			75.0		
	An^−^	88.5			84.6		

*Sn*: sensitivity; *AA*: average accuracy; *OA*: overall accuracy; IC: ion channels; NIC: non-ion channels; VGIC: voltage-gated ion channels; LGIC: ligand-gated ion channels.
